# Editorial: Photodynamic therapy: challenges and innovations for treating cancer

**DOI:** 10.3389/fonc.2024.1526550

**Published:** 2024-12-06

**Authors:** Newton Soares da Silva, Juliana Ferreira-Strixino, Cristina Pacheco-Soares

**Affiliations:** ^1^ Institute of Science and Technology, São Paulo State University (Unesp), São José dos Campos, SP, Brazil; ^2^ Institute of Research and Development (IP&D), Universidade do Vale do Paraiba (UNIVAP), São José dos Campos, SP, Brazil

**Keywords:** photodynamic therapy, cancer, technological innovations, immunological interactions, synergistic therapies

## Introduction

The treatment and prognosis of cancers remain challenging for researchers and patients, which makes it necessary to study alternative therapies as primary or adjuvant treatments to currently existing methods. Photodynamic therapy (PDT) has emerged as a promising approach for treating several types of cancer due to its selective targeting capacity and light-induced cytotoxic effects. This Research Topic, *Photodynamic Therapy: Challenges and Innovations for Treating Cancer*, aims to explore the persistent challenges and recent innovations in PDT to improve its clinical efficacy and safety. This collection of manuscripts reflects the collaborative efforts of researchers to better understand the mechanisms of PDT, optimize photosensitizing agents, and advance techniques for the delivery and activation of the therapy.

PDT is a key tool to enhance or optimize the treatment of resistant and obstructive tumors, such as advanced colorectal and esophageal cancer. The association of PDT with chemotherapy, anti-inflammatory agents, or technologies such as Photochemical Internalization (PCI) shows that PDT promotes tumor destruction and modulates the immune system by recruiting immune cells and activating defense mechanisms against cancer. A complete review of ongoing and completed clinical trials evaluating the impact of photosensitizers on the immune response after PDT in cancer therapy revealed that a wide variety of photosensitizers are still under investigation, but there is still a lack of evidence on their effects on immune cells (Fan et al., Dudzik et al.). PCI is a drug delivery system based on PDT. Longva et al. demonstrated that PCI, together with the vascular targeting toxin VEGF121/rGel, induces immune-mediated cell death. On the other hand, the association of liposomes (ICG-Lipo) with the chemotherapy drug paclitaxel (PTX) demonstrated that this new molecule accumulates specifically in tumors due to the characteristics of the liposomes, significantly increasing the action of this treatment (Ishizuka et al.).

Ferroptosis is a type of programmed cell death that plays an important role in cancer progression. Surufatinib has a good systemic effect and long duration, which can be used to inhibit tumor cell proliferation after PDT. Surufatinib induces ferroptosis in malignant tumor cells; therefore, the combination of saracatinib and PDT treatments may increase the effect of treatments by overcoming tumor resistance and prolonging patient survival (Huang et al.).

An study about esophageal cancer cells after AlPcS4Cl-mediated PDT demonstrated that this therapy reduced cell viability, induced cytotoxicity, cell cycle arrest in the G0/G1 phase, and DNA double-strand break (DSB). These studies confirm cell death by apoptosis after AlPcS4Cl-mediated PDT (Didamson et al.), which is beneficial to patients as it minimizes side effects of classic treatments such as chemotherapy. The association of PDT with new technologies, such as robotic surgery and nanotechnology, has allowed the advancement of minimally invasive surgery in oncology. Huang et al. describe a case of colorectal cancer with intestinal obstruction and stenosis. After four sessions of PDT, the patient’s intestinal lumen was unobstructed, and the tumor lesion regressed. Subsequently, the tumor was completely resected with good surgical results. Thus, the combination of PDT and fluorescence-assisted robotic surgery may offer new therapeutic alternatives for patients with advanced colon cancer.

Hao et al. developed a nanoparticle (CPT-TK-Pa/Pt) that increases the production of reactive oxygen species to enhance and assist the effects of PDT against colon tumors. After irradiation with a 660 nm laser, the CPT-TK-Pa/Pt nanoparticle demonstrated an intensified cytotoxic effect and inhibited the proliferation of tumor cells.

The studies presented in this Research Topic reaffirm the value of PDT as a versatile and promising strategy in fighting against cancer. The use of optimized photosensitizers advances in administration techniques, and the combination of technological innovations position PDT as an increasingly relevant modality capable of acting synergistically with other therapies. Despite advances, challenges such as the standardization of photosensitizers and understanding their immunological interactions still require further investigation. Continued research in this field could transform PDT into a practical and less invasive complementary treatment for a broader spectrum of cancers, offering alternatives for patients with resistant and complex tumors.

